# Does It Matter Where You Live? Rural–Urban Context Among Women Entrepreneurs in Pakistan

**DOI:** 10.3389/fpsyg.2022.827634

**Published:** 2022-03-03

**Authors:** Said Muhammad, Kong Ximei

**Affiliations:** ^1^School of Business, Zhengzhou University, Zhengzhou, China; ^2^Yunus Social Business Centre, School of Business, Zhengzhou University, Zhengzhou, China

**Keywords:** women entrepreneurs, rural–urban context, socio-economic factors, regional development, family financial position

## Abstract

Entrepreneurship is considered as one of the strategies for economic and regional development. In particular, women entrepreneurs engaged in different geographic locations, where their characteristics and business factors are different in each location. This study examines home-based women entrepreneurs in Pakistan in relation to their place of residence, specifically rural or urban context. Very few studies have considered place of residence as a variable affecting women’s businesses at the household level. This is critical since the business context can exert a major influence on available resources and constraints that affect business viability and sustainability. Data were collected from 504 women entrepreneurs using a survey questionnaire. Descriptive statistics, chi-square test, and binary logistics regression were used to achieve the objectives of the study. The findings revealed important and significant differences based on the rural versus urban context of women entrepreneurs including home ownership, household size, the number of adult family members in the household, family financial position, business record keeping, having a bank account, and type of business. While the binary logistic regression analysis reported adult family members, family financial position, business record keeping, bank account, and beautician business were the significant predictors of the women entrepreneurs’ rural–urban model. The findings offer implications for policymakers, funders, bank/financial institutions, and non-governmental organizations for increasing women’s entrepreneurship, empowerment, and income equality in developing countries.

## Introduction

Entrepreneurship has been considered a crucial vehicle for the economic development regionally (Audretsch and Belitski, 2021) and worldwide. In Pakistan, entrepreneurship is helping to change the traditional values and status quo of the society (Wrigley-Asante, 2011) with women entrepreneurs at the forefront of these changes (Yunis et al., 2020). Yet, in the developing countries most women entrepreneurs are poor and often engaged in rural business activities (Muhammad et al., 2020), even though similar results have been reported for women’s rural businesses in the United States (Conroy et al., 2021). Although entrepreneurship has been considered a sector dominated by men (Syed, 2010), studies have revealed the significance of the women’s contributions to economic empowerment through entrepreneurship (Jabeen et al., 2020). Women’s entrepreneurship has played a significant role in generating jobs (Nahid et al., 2019), social inclusion (Kemp and Berkovitch, 2020), gender equality (Ul Hassan and Naz, 2020), wellbeing (Lepeley et al., 2020; Muhammad et al., 2021a), and increasing living standards by alleviating poverty (Nasir et al., 2019).

This study focuses on the environment of women’s entrepreneurship in a developing context. Empirical studies at the microlevel of social capital are still a relatively new area of research in the developing countries, mainly due to a lack of disaggregated data (Saqib et al., 2016). Most studies do not focus on the context of entrepreneurship (Yunis et al., 2020). This study delves into the microlevel to examine rural and urban entrepreneurship environments. Urban environments would appear to be more conducive to women’s businesses, but more work is needed to understand and to identify specific factors affecting the viability and sustainability (Jabeen et al., 2020) of women’s rural and urban, home-based entrepreneurship.

Our paper proceeds as follows. First, we review the institutional and religious context navigated by women entrepreneurs in Pakistan. Next, we review the literature on the rural and urban environments of women’s businesses in a developing context. Then we explain our sampling procedure, questionnaire development, and analytic strategy. Next, we present our results comparing rural and urban women’s businesses in relation to socio-demographic variables. Finally, we discuss the results in relation to existing literature, offer suggestions for additional research, and discuss policy implications in our conclusion.

### Institutional and Religious Context of Pakistan

Islam does not restrict women’s participation in business and other economic activities as per *Sharia* (Muhammad and Ximei, 2020). Pakistan is the second-most populous Muslim country in the world. Pakistan has a Muslim population of 96.4% with women representing 49.2% of the population to (Yunis et al., 2019). But women’s participation in entrepreneurial activities is very low as compared to that of men (Jabeen et al., 2020). As reported by World Bank (2019) a large share of Pakistan’s greatest asset, it population, is being wasted by excluding women from the labor force. Due to this unequal development of geographical regions and ethnic origins have an impact on the socio-economic development of Pakistan (Roomi et al., 2018). Women do not have equal rights as business sectors is considered a male dominant profession (Dinar, 2020). Gender discrimination is still present in the labor force both socially and culturally (Roomi et al., 2018). The societal beliefs and gender biases not only affect the businesses of women entrepreneurs, but also create barriers to receiving maximum benefits from their work engagements. According to Roomi and Harrison (2010) and Muhammad et al. (2020), In Pakistan, women are not always allowed to go out of their homes to participate in mixed-gender programs. There are various reasons for restricting women’s free mobility (e.g., lack of education, managing work–life balance, personal safety issues, and most importantly, socio-cultural practices) representing an impediment for women entrepreneurs.

Because of the dominance of the socio-cultural and religious norms, women are considered as housewives and custodians of family honor (Muhammad et al., 2020). The impelling societal, cultural, and conservative norms and practices are widespread across the region. However, legislation follows the Islamic teaching, but the social setting is often induced by cultural and pre-Islamic tribal trends (Pakeeza, 2015). Men are provided with better education and skills while women are relegated to practicing domestic skills in order to be useful at home. This discrimination leads to the economic and social dependency of women in the circle (Yunis et al., 2019), making women subordinate to men in the society. Furthermore, it also reduces their social status and roles as producers and providers. Women are no longer willing to tolerate social discriminatory practices. By operating their business ventures from home, these women entrepreneurs can enhance their individual and family wellbeing with in their cultural and Islamic context.

To make Pakistan a “welfare state,” the government has started “*Ehsaas*” cash program in April 2019 to invest in people by uplifting the less developed areas (Government of Pakistan, 2019). The Pakistani government, by providing managerial and technical skill promotes self-employment opportunities for women who wants to start a business to contribute significantly toward socio-economic development (Ramadani, 2015). Moreover, by promoting several schemes such as easy access to digital services, and financial inclusion by enhancing women’s socio-economic empowerment with a special focus on poverty alleviation. This could be an opportunity to offer additional assistance to these women informal entrepreneurs. The socio-economic and political changes can bring about new social structures that can have a positive effect on women’s freedom of movement enhancing their empowerment (Roomi, 2013).

Furthermore, women’s profitable economic engagements promotes financial independence to empower them while contributing more to the country economic development (Anggadwita et al., 2017). The dominant cultural values make Pakistan differ from the rest of the world. As a result of these cultural limitations, women have no option but to run their businesses from their homes. While on the other side, establishing a business at home is not simple since it requires permission and consultation with family members. Most informal businesswomen in Pakistan are engaged in own-account ventures instead of family businesses, based on the need to reconcile the social demands and expectations of the family as gendered constraints (Chant, 2014). The context of entrepreneurship needs to be examined further in a developing context to know the rural–urban differences by considering women entrepreneurial socio-economic factors, business characteristics, and products or services provided. Most literature has focused either on rural *or* urban women entrepreneurs. While it differs from country to country and within the developed and developing nations.

Therefore, the study has two major objectives. First, to examine the rural–urban differences by taking the socio-economic and business factors of women entrepreneurs. Second, to investigate the impact of socio-economic factors and products/services provided in relation to the location of women entrepreneurs. The study examines both contexts, rural and urban, simultaneously by taking women home-based entrepreneurship in the informal sectors as a case. This is particularly important in a developing context where the boundaries between rural and urban environments remain discernible with respect to geography and lifestyle.

## Literature Review

Entrepreneurship is socially bound but it also has a spatial and geographical contexts as well (Welter, 2011). The context of entrepreneurship is a critical dimension that has received lesser emphasis in the literature than other entrepreneurship variables (Yunis et al., 2020). One spatial aspect that has been explored is the household and family (Mirchandani, 1999) such that women often select the most convenient business site, the home. This is particularly noteworthy in a developing context like Pakistan where women often have limited or no other options than a home-based business because of restrictions on women’s behavior due to socio-cultural norms. However, these socio-cultural norms are not uniform across the whole country. Therefore, our focus is on the rural–urban differences in women’s home-based entrepreneurship in one of the province Khyber Pakhtunkhwa, Pakistan. Rural–urban differences are known to exist in this province including differences in education level, house construction, health services, roads, water supply, sanitation, etc. virtually every area that was investigated (Rahman et al., 2011). However, it is unclear how such overall differences affect rural–urban women’s entrepreneurship.

Research has found that economic activities in urban locations generate higher incomes as compared to rural venues (Shabbir and Di Gregorio, 1996; Yu and Artz, 2019). The rural–urban context was studied in Vietnam, showing that remittances improve rural welfare, but do little to reduce inequality (Bui and Imai, 2019). Further, public policy recommendations included increased access to education for the rural poor while supporting self-employed as a strategy to increase income (Bui and Imai, 2019). Minh Chau (2020), using field work from Vietnam, reported on home-based businesses among rural households and the ways in which the opportunities and uncertainties of marketization. The strategy of rural households in response to the state’s push toward home-based enterprises is “đa gi năng,” a local term that means “keeping many livelihood options and never putting all eggs in one basket” (Minh Chau, 2020). Another study in Taiwan, China reported that, these activities will help to reduce income inequality in rural areas (Chinn, 1979; Ho, 1979).

Some authors have reported different findings. For example, studies conducted in Burkina (Reardon et al., 1992) and Nigeria (Matlon, 1979), found a negative impact on the income distribution in rural communities. The results of a study showed dual impact: on one hand, the poor households depend on these economic activities as they are unskilled but income from these businesses has a favorable impact. While, on the other hand, the rich households depend on the employment in the government sectors contributing to income inequality (Adams, 1994). Spatial inequality and higher education were the two significant factors in inequality (Heltberg, 2003).

When assessing entrepreneurship in a developing context, we need to understand the factors that motivate women to start and run a business (Anwar-Ul-Haq et al., 2014). Several variables that affect entrepreneurship as a career choice have been identified including age (Dary and Kuunibe, 2012; Litsardopoulos et al., 2020), education (Shehu and Abubakar, 2015) marital status (Dary and Kuunibe, 2012), household size (Babulo et al., 2008), family support (Constantinidis et al., 2019), location (Brown et al., 2006; Shehu and Abubakar, 2015), access to markets (Wanyama et al., 2010), access to credit (Kuwornu and Dumayiri, 2014; Tisdell et al., 2020) and social networks (Shehu and Abubakar, 2015). Social networks in literature about livelihood activities have been reported (Scoones, 1998), and in the rural context, social networks are more important as compared to urban settings particularly for securing micro-loans (Tisdell et al., 2020). Networks help in promoting social capital that is essential for women entrepreneurs to facilitate, sustain, access to resources and opportunities (Ayifah et al., 2021).

Urban women have more employment opportunities in both formal and informal sectors thereby increasing the chance of earning a higher income although these effects are not uniform (Hanson et al., 1997). Rural women, on the other hand, have more social and cultural restrictions, fewer job and employment opportunities, lower levels of education (Abrar-ul-haq et al., 2017), and limited access to markets and financing rendering home-based entrepreneurship (Tisdell et al., 2020). These barriers increase the gap between urban and rural women entrepreneurs. The average income values for rural and urban areas show rural–urban disparities, for example, (Cheng et al., 2020) suggesting that, on average, the urban individual’s income and spending is double as compared to rural individual. On the other hand, with relation to expenditures, the cost of living in rural areas is lower due to the consumption home produced eatables (Van Cao and Akita, 2008).

It seems clear that, in terms of income levels (Novotný, 2007) and business infrastructure, that urban and rural locations have different features. One study showed that the business environment for women in Pakistan is complex because of socio-cultural, tribal, and religious factors that directly affect women seeking to improve their status (Goheer, 2003; Anwar-Ul-Haq et al., 2014; Roomi et al., 2018). Because of differential investment levels and low turnover, rural women find it difficult to reach new markets (Gautam and Andersen, 2016). Many women also have families that can affect their work involvement (Xheneti et al., 2019). Furthermore, full time jobs in the corporate sector along with family care responsibilities become sources of stress for women (Edwards and Field-Hendrey, 2002; Gopalan et al., 2020). While formal sector demands for labor are not as high as those in the developed countries. It means that external social factors can also play a role in shaping socialization processes that can reduce inequality of opportunities and classism. Both formal and informal institutional elements can affect women’s perceptions of entrepreneurial opportunities. Most of the women in Pakistan are engaged in the agricultural sector, followed by services then industry (Adams, 1994; UNDP, 2007). These women are not highly educated and lack necessary skills that are required in the formal sector.

The findings of (Muhammad et al., 2021c) reported that urban women have an average and above-average family financial position while most of the rural women entrepreneurs were belong to a lower than average family financial position. However, the family’s financial position also effects the choices of women entrepreneurs, especially when their family has a strong financial base to support them financially in the startup and meeting their running entrepreneurial expenditures (Muhammad et al., 2021b). The family financial position in these studies was not measured in absolute values but were reported by the respondents that how they see their family financial position by selecting in the below average, average, and above-average family financial position. Furthermore, home-based businesses are the primary option for women to earn an income (Sultana et al., 2020) thereby contributing to their family’s financial wellbeing (Haughton and Vijverberg, 2002; Muhammad et al., 2021a). Recent work has termed this the “multiplier effect” of women’s entrepreneurship (Lepeley, 2020) that ultimately leads to better education and health of their children and families.

Home-based businesses offer a work arrangement that has several advantages such as flexible hours, decision autonomy, financial benefits associated with not having to travel to do business in other geographic locations (Christensen, 1987), and lower levels of business exit (Madanoglu et al., 2020). This convenience of working at home will motivate and help women to reduce the stresses associated with more formal work engagements. As a result of easy access to information and more business opportunities, urban women are in a better position to grow their businesses (Syed, 2010). And, because women are restricted to their homes (Yunis et al., 2020), restrictions on free social mobility underestimate women’s ability to become entrepreneurs and to reap the benefits from starting a business (Syed, 2010). Furthermore, the other reasons of free mobility restrictions are socio-cultural restrictions (Sultana et al., 2020) and transportation problems that are more severe for rural women. These barriers restrict women’s professional choices because they can only deal with other women in positions such as beautician, tailoring, and textiles. The involvement of women in entrepreneurial activities is an attempt to enhance family empowerment in the rural and urban areas, promoting economic growth leading to socio-economic development and increased equality.

## Materials and Methods

### Material and Measurement

This study examines the geographic location, rural or urban, of women entrepreneurs in Pakistan. The dynamic nature of participation of women entrepreneurs in Pakistan’s informal sector was explored using a survey conducted from June to August of 2019. The data were gathered from the rural and urban location of the district Mardan, Khyber Pakhtunkhwa province. This district was purposively selected because of its population (2.3 million) and its central location in the Khyber Pakhtunkhwa province (Saqib et al., 2016). The population have been divided into rural–urban on the basis of geographic location. In Pakistan 63 percent of the population resides in rural areas (PBS, 2017–18). Respondents were defined as women who were engaged in any sort of home-based economic activities (i.e., women entrepreneurs who were conducting business from their homes).

### Sampling Technique

The sample was selected using a proportional allocation distribution of the rural–urban areas to insure adequate representativeness. Information was collected from the local government and a survey list prepared by the researcher about women entrepreneurs. We identified 2000 women’s businesses engaged in diverse economic activities in rural–urban geographical locations. After applying the (Yamane, 1967) formula, 391 women entrepreneurs as a representative sample by considering percent margin of error. The derived sample estimate was 391, but the actual sample exceeded the estimate, resulting in 504 respondents.

### Data Collection

Questionnaires were used to collect quantitative data from 504 participants. The survey included open- and closed-ended questions including socio-economic and other business factors. Because of the low level of literacy and subject matter sensitivity, face-to-face interviews were essential. Interviewer administered questionnaires were used for those participants who were less or uneducated (Buor, 2004; Goldstein et al., 2018). Educated women entrepreneurs were permitted to complete the questionnaire by themselves. Pashto is the local language that is spoken, written, and read. The questionnaire was developed in English and translated into “Pashto.” Because of cultural norms regarding male–female interactions, women assistants with master’s degrees were hired and trained to collect the data. These assistants were closely supervised by the researcher to minimize bias. Respondents represented eight types of business including: dairy, cloth, groceries, stitching, hand embroidery, beauticians, cosmeticians, and meat business based on the reconnaissance survey.

### Data Analysis

The Data checked and prepared before analysis. After checking, the dataset was analyzed using IBM SPSS 27 software. Descriptive statistics was used for the coded description, frequencies, percentages, means, and standard deviations. Bivariate analysis techniques were used to test association and predictors group differences on the basis of urban–rural geographic location of the respondents. In multivariate analysis, binary logistic regression Equation 1, was employed on those predictors which were found significant in chi-square test. Binary logistics model can better evaluate the relationship of binary dependent and other independent variables (Stock and Watson, 2015).


Equation 1 $$\begin{array}{l} Y_{i}=\log\left[\frac{p}{1-p}\right]=logY=\beta_{0}+\beta_{1}X_{1}+\\ \beta_{2}X_{2}+\beta_{3}X_{3}+\beta_{4}X_{4}+\\ \beta_{5}X_{5}+\beta_{6}X_{6}+\beta_{7}X_{7}+\mu \end{array}$$

Where; $Y_{i}$= 0 for urban and 1 for rural; $\beta_{0}$= constant; $\beta_{1}$= coefficient of the *i*th independent variables and $X_{1}$= independent variables where *i* = 1–7; and μ=error term.

## Results

### Descriptive Analysis

Summary statistics are shown in Table 1. Descriptive results of the study socio-economic demographics and business factors are reported in Table 1. The dependent variable geographic location was coded as 0 for urban and 1 for rural areas. Nearly 41% of the respondents lived in urban areas, while the rest were reported that they resided in a rural location.

**Table 1:** Descriptive analysis of variables (*n* = 504).

**Variables**	**Variables description and coding**	***n***	**%**	**Mean**	***SD***
**Dependent variable**					
Geographic Location	0 = Urban	206	40.9	–	–
	1 = Rural	298	59.1	–	–
**Independent variables**					
Age	Age of respondents in a year of groups	504	–	36.43	8.88
Education	Education as years of schooling	504	–	4.96	5.11
Marital status	0 = Single	88	17.5	–	–
	1 = Married	416	82.5	–	–
Homeownership	0 = No	158	31.3	–	–
	1 = Yes	346	68.7	–	–
Monthly Income	up to 8,000	190	37.7	–	–
	8,001–15,000	144	28.6	–	–
	15,001–25,000	87	17.3	–	–
	More than 25,000	83	16.5	–	–
Household size	Total members in the family	504	–	6.95	2.28
Adult Family Members	Total adult family members	504	–	4.08	2.03
Family financial position	1 = Lower than average	159	31.5	–	–
	2 = Average	201	39.9	–	–
	3 = Better than average	144	28.6	–	–
Decision-Making	0 = Herself	267	53.0	–	–
	1 = Family	210	41.7	–	–
	2 = Consultation with friends	27	5.4	–	–
Business Experience	Experience in years	504	–	8.32	1.04
Type of Business	0 = Manufacturing	92	18.3	–	–
	1 = Trade	242	48.0	–	–
	2 = Service	170	33.7	–	–
Form of Business ownership	0 = Sole Proprietorship	442	87.7	–	–
	1 = Partnership	62	12.3	–	–
Other Business	0 = No Business	434	86.1	–	–
	1 = One or More	70	13.9	–	–
Book keeping	0 = No	318	63.1	–	–
	1 = Yes	186	36.9	–	–
Bank account	0 = No	386	76.6	–	–
	1 = Yes	118	23.4	–	–
Products/Services	0 = Dairy	46	09.1	–	–
	1 = Cloth	113	22.4	–	–
	2 = Grocery	106	21.0	–	–
	3 = Stitching	83	16.5	–	–
	4 = Hand Embroidery	59	11.7	–	–
	5 = Beautician	28	5.6	–	–
	6 = Cosmetics	42	8.3	–	–
	7 = Meat	27	5.4	–	–

Author’s calculations—SD standard deviation.

The mean age of the participants was 36 years (*SD* = 8.88). Education, measured as years of schooling, showed that the average education of women entrepreneurs was reported 4.96 years (*SD* = 5.11). Most of the women entrepreneurs were married (82.5%) which is in line with a study conducted in Israel (Heilbrunn and Davidovitch, 2011). About 68.7% reported owning their home. Most of participants have income less than PKR 8000 while 28.6% of the entrepreneurs reported incomes in the range of PKR 8001–15,000. The average of household size was 7 members (*SD* = 2.28), while the number of adult family members averaged 4 members (*SD* = 2.03). More than half of women entrepreneurs reported that they made their own business decisions, another 41.7% reported consultation with family members, and a small minority reported consultation with friends in making business decisions. Women averaged 8.32 years of business experience (*SD* = 1.04). Nearly half of the women participants were engaged in trade business activities. Most of businesses were owned by a single owner and having no other business than the business they were engaged. 63.1% of the respondents reported that they have no bookkeeping record of their transaction and 76.6% have not bank account in the banks. The products/services offered by the women entrepreneurs included cloth (22.4%) followed by grocery (21%) with the remainder distributed among other businesses.

### Bivariate Analysis

A series of cross-tabulations (chi-square test) of geographic location by predictors were estimated first. The results of chi-square test of association were reported in Table 2. Home ownership, household size, adult family members, products offered, business record keeping, maintaining a bank account, and family financial position were found to be statistically significant. About 30% of the entrepreneurs were between the ages of 30–35 years. This is an indication of mostly young people are inclined toward entrepreneurship. The age of rural and urban women entrepreneurs was nearly the same. Most of participants were illiterate, but women entrepreneurs with higher educational attainment (beyond secondary school certificates) were predominantly from urban locations. Considering marital status, nearly 18% of the respondents were single while 82% of women entrepreneurs in rural locations were married. This might suggest that women’s engagement in economic activities was necessity-based, serving as a means of supporting their families and children. Home ownership was significant with 74.3% of the urban respondents and 64.8% of the rural reporting homeownership.

**Table 2:** Bivariate association of geographic location and independent variables.

**Variables**	$\chi^{2}$	**Geographic location**	
		Urban *n* = 206	Rural *n* = 298
**Age (Year)**	0.14	**f (%)**	**f (%)**
18–29		40 (19.4)	58 (19.5)
30–35		62 (30.1)	89 (29.9)
36–42		61 (29.6)	85 (28.5)
43 and above		43 (20.9)	66 (22.1)
**Education**	4.91		
Illiterate		78 (37.9)	133 (44.6)
Primary School		37 (18.0)	51 (17.1)
Secondary School certificate		51 (24.8)	76 (25.5)
Higher than Secondary School		40 (19.4)	38 (12.8)
**Marital Status**	2.82		
Single		43 (20.9)	45 (15.1)
Married		163 (79.1)	253 (82.5)
**Home Ownership**	5.11^*^		
No Ownership		53 (25.7)	105 (35.2)
Ownership		153 (74.3)	195 (64.8)
**Monthly Income**	3.40		
Up to PKR 8000		86 (41.7)	104 (34.9)
8,001–15,000		52 (25.2)	92 (30.9)
15,001–25,000		37 (18.0)	50 (16.8)
More than 25,000		31 (15.0)	52 (17.4)
**Household Size**	10.15^*^		
2–5		57 (27.7)	94 (31.5)
6–7		72 (35.0)	87 (29.2)
8–9		56 (27.2)	61 (20.5)
10–15		21 (10.2)	56 (18.8)
**Adult Family Members**	8.30^*^		
Up to 2		55 (26.7)	105 (35.5)
3–5		103 (50.0)	111 (37.2)
6 and above		48 (23.3)	82 (27.5)
**Family financial position**	51.03^**^		
Lower than average		30 (14.6)	129 (43.3)
Average		93 (45.1)	108 (36.2)
Better than average		83 (40.3)	61 (20.5)
**Decision-Making**	1.65		
Herself		105 (51.0)	162 (54.4)
Family		92 (44.7)	118 (39.6)
Consultation with friends		09 (04.4)	18 (06.0)
**Business Experience (years)**	1.60		
Up to 3		47 (22.8)	65 (21.8)
4–6		66 (32.0)	83 (27.9)
7–13		58 (28.2)	90 (30.2)
14–30		35 (17.0)	60 (20.1)
**Type of Business**	1.24		
Manufacturing Business		36 (17.5)	56 (18.8)
Trade Business		105 (51.0)	137 (46.0)
Services		65 (31.6)	105 (35.2)
**Form of Business ownership**	2.44		
Sole Proprietorship		175 (85.0)	267 (89.6)
Partnership		31 (15.0)	31 (10.4)
**Other Business**	0.13		
No Business		176 (85.4)	258 (86.6)
One or More		30 (14.6)	40 (13.4)
**Book keeping**	17.03^**^		
No		108 (52.4)	210 (70.5)
Yes		98 (47.6)	88 (29.5)
**Bank account**	17.90^**^		
No		138 (67.0)	248 (83.2)
Yes		68 (33.0)	50 (16.8)
**Products/Services**	18.43^*^		
Dairy		17 (08.3)	29 (09.7)
Cloth		48 (23.3)	65 (21.8)
Grocery		44 (21.4)	62 (20.8)
Stitching		27 (13.1)	56 (18.8)
Hand Embroidery		29 (14.1)	30 (10.1)
Beautician		20 (09.7)	08 (02.7)
Cosmetics		13 (06.3)	29 (09.7)
Meat		08 (03.9)	19 (06.4)

Source: Field survey 2019,

* *p* < 0.05 level.

** *p* < 0.01.

The significant result of household size revealed that 6–7 family members was the average in urban locations while 10–15 members of household size 18.8% belong to rural location. 50% of the adult family members variable comprise of 3–5 members while more than 6 members, 27.5% belong to rural geographic location. This result is statistically significant. 41.7% of the respondents in the urban have monthly income up to PKR. 8,000. Considering type of business, 51% and 46% of the entrepreneurs in the urban and rural locations respectively, were engaged in the trade business while most of respondents are sole proprietors with no other business. The cloth business percentage in the urban and rural are the almost the same followed by grocery business. 52.4% and 70.5% in the urban and rural, respectively, have no proper business record. In addition, the respondents who have kept business record, 47.6% belong to urban location.

Most of the women entrepreneurs did not have a bank account, although 33% of respondents living in an urban area did have bank account versus 17% of rural women. Family financial position played an important role in supporting women entrepreneurs: 45.1% of the urban entrepreneurs reported an average family financial position, but 43.3% of the rural entrepreneurs reported a lower-than-average family financial position.

### Multivariate Analysis

The multivariate analysis indicate the relationship showed in the chi-square test still retains while controlling the effects of other variables. Since the dependent variable was dichotomous (rural or urban), logistic regression was used to predict the probability of whether the occurrence of an event or not is the nature of dependent variable—categorical having only two possibilities that the location of the entrepreneurs “urban or rural,” with a value of 0 and 1, respectively. As shown in the Table 3, adult family members, products provided, business record, bank account, and family financial position were the significant predictors of geographic location. Except for these, all other variables in the model were insignificant. Furthermore, we have reported the goodness of fit test (Hosmer–Lemeshow Test) along with values of Cox and Snell and Nagelkerke *R*^2^. According to the model, chi-square was 8.29 (0.405) indicating that the model fits the data well.

**Table 3:** Binary logistic regression analysis.

Variables	Coefficient	Standard error	*p*-value	Odd Ratio (95%CI)
**Home Ownership**				
No Ownership^®^	1.00			
Ownership	0.351	0.266	0.186	1.42 (0.84–2.39)
**Household Size**				
2–5^®^	1.00			
6–7	0.067	0.270	0.805	0.81 (0.55–1.59)
8–9	−0.564	0.30	0.60	0.57 (0.32–1.02)
10–15	0.666	0.37	0.07	1.95 (0.94–4.01)
**Adult Family Members**				
Up to 2^®^	1.00			
3–5	−0.562	0.25	0.03^*^	0.57 (0.35–0.94)
6 and above	−0.182	0.30	0.55	0.83 (0.46–1.50)
**Family financial position**				
Lower than average^®^	1.00			
Average	−1.53	0.29	0.000^**^	0.22 (0.12–0.39)
Better than average	−2.05	0.34	0.000^**^	0.13 (0.07–0.25)
**Book keeping**				
No^®^	1.00			
Yes	−0.645	0.24	000^**^	0.52 (0.33–0.85)
**Bank account**				
No^®^	1.00			
Yes	−0.673	0.26	0.01^*^	0.51 (0.30–0.86)
**Products/Services**				
Dairy^®^	1.00			
Cloth	0.31	0.41	0.45	1.37 (0.61–3.08)
Grocery	−0.10	0.41	0.80	0.90 (0.35–0.94)
Stitching	0.05	0.42	0.90	1.06 (0.46–2.42)
Hand Embroidery	−0.65	0.45	0.14	0.52 (0.22–1.25)
Beautician	−1.32	0.59	0.02^*^	0.27 (0.08–0.85)
Cosmetics	0.43	0.52	0.39	1.54 (0.58–4.11)
Meat	0.70	0.56	0.21	2.01 (0.67–6.08)
Constant	2.182	0.453	0.000^**^	
−2 Log Likelihood		567.62		
Nagelkerke *R*^2^		0.273		
Hosmer and Lemeshow Test $\chi^{2}$ (*p*-value)		8.29 (0.405)		

CI, Confidence interval; ® = Reference group,

* *p* < 0.05.

** *p* < 0.01.

The odds ratio interpretation provides the association between binary dependent and other categorical or continuous predictors. The odds ratio of the first significant variable, adult family members (3–5), is 0.57 times less likely to fall in the urban as compared to rural location. The second significant variable is beautician. The odds ratio of beautician is 0.27 times more likely to report in the urban location as compared to dairy products. Those entrepreneurs who have business records and a bank account have odds ratios of 0.52 and 0.51, respectively. Furthermore, respondents who do not keep business records and had no bank account were more likely to reside in a rural location. Further, the odds ratio for “average” and for “better than average” family financial position is 0.22 and 0.13, respectively. The odds ratio showed that, as a reference to lower-than-average category of family financial position, the average and better than average categories were more likely to reside in urban locations.

## Discussion

The Pakistan labor market is not fully developed so women’s businesses act as a crucial source of income and employment for families. The mean age of the study participants is lower than a study conducted in Mauritius (Kasseeah, 2014), but suggests the attraction of younger women entrepreneurs toward home-based businesses. Similar age findings were reported in another study conducted in rural Pakistan (Adams, 1994). The high percentage of married women showed the women’s entry (Parker, 2008) into home-based businesses might represent a survival income stream for their families. Home-based business can be a source of motivation for women who can manage a business as well as family responsibilities. As discussed earlier, in Pakistan, these women adopted businesses for the sake of financial security, consistent with necessity-based entrepreneurship. As stated by Majumder (2020), when it becomes difficult for the household to meet expenditures, women’s businesses come into play to support them. Nevertheless, some of these home-based businesses succeed beyond meeting household expenditures.

A major difference between rural and urban women entrepreneurs was literacy. Although more women entrepreneurs resided in rural areas, those who obtained higher secondary school certificates, mostly lived in urban locations. Education levels have been a consistent difference between for rural and urban areas (Rahman et al., 2011). Years of education is important because education has a significant positive correlation with women’s entrepreneurship (Razmi and Firoozabadi, 2016). There is also a positive relationship between securing and utilizing micro-finance loans and growth of women’s businesses (e.g., Tisdell et al., 2020). Education is a critical intervention point consistent with the UN’s Sustainable Development Goals, women’s entrepreneurship, and regional economic development (Audretsch and Belitski, 2021).

The homes in rural locations have more space that can be used to support business activities. In fact, although the income from these home-based economic activities is low, such businesses have the advantage of saving on rent. Furthermore, the underused capacity in their homes can be used to minimize the costs of their business activities (Edwards and Field-Hendrey, 2002). They can give more time to business and no need to pay transportation cost because men travel from their homes to do business far away from families. The typical household consists of 7 members that can be a strength for managing work–life interactions (Perrons, 2003; Khandelwal and Sehgal, 2018). The larger households are likely families residing in a rural location. The adult family members can encourage (Grünberg and Matei, 2020) women members to play a role in family financial status particularly in the urban locations where living expenses are higher.

Decision-making has an impact on the growth of women’s businesses (Noor et al., 2021). These economic activities will empower women to be more independent decision-making. The involvement of family and friends in decision-making helps to refine ideas but such input can also influence the decisions of women entrepreneurs and timely decisions get implemented. These businesses can also be converted into partnerships with in the families in both urban and rural areas (Tahir et al., 2018). The urban areas have more job opportunities in both the formal and informal sectors. Furthermore, it also provides a conducive environment for business startups and expansion of existing entrepreneurial activities. The income from these jobs and businesses helps in enhancing their family financial status.

Because these entrepreneurial businesses are informal and small in size, women have no formal set of books of accounts. This might be explained in a couple of ways. Women might lack the education and training needed to maintain formal bookkeeping records. The education has the main role to record proper books of account while the findings of this study reported that more than 40% of the women are not formally educated, which can be one of the reasons. However, their businesses are operating on a small scale and are homo-based that could be another reason of not record their business transactions. Women entrepreneurs might be reluctant to seek the help of other family members since this might compromise business control and autonomy. Women entrepreneurs have power since they recognize the positive contributions that they are making (Jabeen et al., 2020). A study (Gathuru and Rukaria, 2021) conducted in Kenya showed that book keeping skills have a significant effect in order to enhance the effectiveness of financial control. Furthermore, another study in South Africa by (Myeko and Madikane, 2019), conducted personal interviews reported that small businesses did not implement proper record of their business transactions as they do not understand its importance. Finally, formal record keeping might be threatening because it might jeopardize the informal nature of the business that would require registration and paying taxes.

Banking facilities are not available for many women entrepreneurs particularly those who live in rural locations. The banking sector focuses on opening their branches in the urban areas to get more customers but rural locations also need access to banks. Women entrepreneurs need capital to sustain and grow their businesses. For home-based women entrepreneurs having a bank account is a big step forward. Having access to credit can also assist in enterprise development. More work needs to be done on the credit needs of women entrepreneurs in relation to borrowing needs, payment schedules, the cultural environment, and outcomes to be achieved.

Rural–urban location disparity was found in the products and services provided by women entrepreneurs. The share of the women in business activities is low as compared to male counterparts in Pakistan (Zeb and Ihsan, 2020). Most of the women entrepreneurs were engaged in cloth business and grocery businesses followed by stitching (tailoring) activities. The significance results for beauticians showed that the urban beauticians have more opportunities to expand their businesses.

Clearly, it does matter where women entrepreneurs live. Many differences between rural and urban women entrepreneurs were identified. Although urban women have some distinct advantages over rural women, the fact that all of these businesses were contributing to the welfare of their families for rural and urban women entrepreneurs must be acknowledged. The family embeddedness of women’s entrepreneurship is clearly evident in the developing context of Pakistan. The findings also provide further evidence of the “multiplier effect” of women’s entrepreneurship (Lepeley, 2020). The multiplier effect refers to satisfaction and work–life integration that has an impact not only on women’s wellbeing (Lepeley et al., 2020), but that of their families, households, children, and communities.

### Study Limitations and Future Research

This study was limited to women entrepreneurs who were engaged in informal entrepreneurial activities. Future research should compare women’s businesses with those of men, in both the formal and informal sectors. Since the data were collected from one district, it is very difficult to generalize the results. Women entrepreneurs can also exercise the option of using new digital technologies in their work to generation additional income (Ughetto et al., 2020). Future studies can explore the benefits and difficulties of using new digital technology in the informal sector.

## Conclusion

Over the last decades, rural and urban women are entering into a diverse economic activities such as manufacturing, trade and commerce, and services, although urban women entrepreneurs have more business opportunities than their rural counterparts. These entrepreneurs can provide inspiration for other women in their circles by using the option of partnership with other family members at home. Home-based businesses are an important source of income for women in both urban and rural locations. Home-based businesses provide women opportunities for flexible hours with respect to location and scheduling (Kim and Parker, 2020). Women’s home-based businesses are different from other types of employment because work site is the home itself. This helps to lower the social and fixed costs of running a business.

Home-based business is an attractive choice for women who live in a rural area with small children and/or other disabled family members (Edwards and Field-Hendrey, 2002). At home, women can spend more hours working rather than trying to find work away from home. Further, working outside the home can be a problem for women living in the rural areas, because women have restricted mobility in a patriarchal society like Pakistan where religious and cultural norms are considered to be more dominant in the society. Women entrepreneurs also have to provide for their families, often-termed the dual role of women. Further, women entrepreneurs help to support their families in both urban and rural areas while contributing to regional development and bringing prosperity to the society. Finally, as confirmed by the results, women’s entrepreneurship enhances the family’s financial position.

### Policy Implications

This study has policy and theoretical implications in enhancing women’s financial empowerment in the society. The government should support women’s small business engagements to support family’s livelihoods and stimulate regional development (Anwar-Ul-Haq et al., 2014). In addition, facilitating and developing women’s businesses will assist in transforming these informal entrepreneurs toward formalization. This will increase tax revenues for the government. Further, women should be protected from all kinds of harassment thereby making the environment more friendly for their mobility necessary for conducting their business in order to enhance their family’s financial status. The banking sector should focus on expanding their network to rural areas, particularly the micro-financial institutions (Tisdell et al., 2020). Extending financial reach will help to provide timely financing, but will also provide bank accounts to women previously lacked them. Sound financing and saving habits particularly in the rural locations will benefit education, health, and wellbeing of women entrepreneurs and their families by contributing to economic growth.

The theoretical contribution derived from the study of women entrepreneurs in the developing context is the “multiplier effect” (Lepeley, 2020), indicating women’s extensive contributions to their households, families, job creation and overall economic and societal development through wellbeing. These business engagements can motivate women entrepreneurs to save, invest for financial security, and increase wellbeing. In the patriarchal context, which influences the ideology of these women entrepreneurs, a comprehensive gendered approach to entrepreneurial support will result in an engaging entrepreneurial development policy and practice.

## Data Availability Statement

The raw data supporting the conclusions of this article will be made available by the authors, without undue reservation.

## Ethics Statement

The studies involving human participants were reviewed and approved by Academic Committee Zhengzhou University. The patients/participants provided their written informed consent to participate in this study.

## Author Contributions

The authors listed have made a substantial, direct, and intellectual contribution to the work and approved it for publication.

## Conflict of Interest

The authors declare that the research was conducted in the absence of any commercial or financial relationships that could be construed as a potential conflict of interest.

## Publisher’s Note

All claims expressed in this article are solely those of the authors and do not necessarily represent those of their affiliated organizations, or those of the publisher, the editors and the reviewers. Any product that may be evaluated in this article, or claim that may be made by its manufacturer, is not guaranteed or endorsed by the publisher.
